# Assessment of Time-Dependent Hydration Products in Olivine-Substituted Cement Mortars

**DOI:** 10.3390/ma18174212

**Published:** 2025-09-08

**Authors:** Yusuf Tahir Altuncı, Cenk Öcal

**Affiliations:** 1 Department of Construction, Vocational School of Technical Sciences, Isparta University of Applied Science, Isparta 32260, Turkey; 2 Department of Civil Engineering, Faculty of Technology, Isparta University of Applied Science, Isparta 32260, Turkey; cenkocal@isparta.edu.tr

**Keywords:** olivine substitution, cement mortar, hydration mechanisms, compressive strength, CO_2_ sequestration

## Abstract

It is known that approximately 8% of atmospheric carbon dioxide (CO_2_) emissions originate from cement production. Consequently, there is ongoing rapid research into environmentally friendly and alternative materials that could substitute for cement. Olivine [(Mg, Fe)_2_SiO_4_] is an abundant mineral in the Earth’s crust that facilitates CO_2_ sequestration due to its high solubility. This study investigates the effects of hydration mechanisms in olivine-substituted cement mortars on their compressive strength, microstructural characteristics, and physical properties. For this purpose, standard cement mortars were produced using CEM IV 32.5 N-type cement with olivine substitution rates of 0%, 10%, and 20%. The compressive strength of the specimens was initially determined at 7, 28, and 90 days. Subsequently, the hydration mechanisms at 7, 28, and 90 days were characterized using X-ray Diffraction (XRD), Fourier Transform Infrared Spectroscopy (FT-IR), Differential Thermal Analysis/Thermogravimetric Analysis (DTA/TG), and Scanning Electron Microscopy-Energy Dispersive Spectroscopy (SEM-EDS). The results demonstrated that the 10% substitution rate complies with the BS EN 196-1 standard, and olivine can be substituted for CEM IV type cement up to 10% without requiring calcination.

## 1. Introduction

The housing demand arising from population growth is the primary reason why cement is the second most used material after water [1]. Global cement production averages 4 billion tons annually, with reports indicating that each unit of cement production generates an equivalent unit of CO_2_ emissions into the atmosphere [2]. Furthermore, it has been reported that approximately 8% of anthropogenic atmospheric CO_2_ emissions originate from cement production [3]. The processes involved in cement production contribute to climate change and global warming [4,5,6]. It is imperative to implement measures against these factors that trigger global warming [7,8]. One such measure is minimizing CO_2_ emissions released during cement production [9,10,11,12,13]. There is ongoing intensive research into alternative raw materials that could substitute clinker, the main raw material, without requiring exposure to high temperatures, and can be used in their natural state [14].

Olivine is one of these alternative minerals abundant in the earth and is highly suitable for CO_2_ sequestration due to its high solubility [15]. It is rich in iron in its chemical composition, and the other high compound in its composition is silicium dioxide (SiO_2_) [16]. Olivine resources on Earth are known to be abundant enough to decompose all anthropogenic emissions for the next 1000 years [17]. It is reported in the literature that the use of olivine aggregates in cement and lime mortars provides environmental benefits by sequestering CO_2_ and converting it to magnesium carbonate (MgCO_3_) [18].

A literature review was conducted by entering the formula “(AB = (olivine) and AB = (cement)) or (TI = (olivine) and TI = (cement)) or (AK = (olivine) and AK = (cement))” into the Web of Science search tab to identify significant studies on the use of olivine powder as a cement replacement material. The resulting 104 studies were individually examined, and 10 research articles within the scope of the investigation were listed. A summary of these studies is presented in Table 1.

In the literature, hydration reactions have been examined in numerous studies, where raw materials such as metakaolin [27,28], graphene [29,30], zeolite [31], calcined clay [32,33], limestone [32,33,34,35], pumice [36], fly ash [37], and diatomite [38] were used as cement replacement materials. However, as evident from Table 1 and the literature review, there are no studies investigating hydration reactions where olivine minerals are used as cement-replacement materials [39,40,41,42,43,44,45,46,47,48,49,50,51,52,53,54,55,56,57,58,59,60,61,62,63,64,65,66,67,68,69,70,71,72,73,74,75,76,77,78,79,80,81,82,83].

Therefore, this study investigates the effects of hydration mechanisms in olivine-substituted cement mortars on compressive strength, microstructural, and physical properties. For this purpose, standard cement mortars were produced by substituting olivine at rates of 0%, 10%, and 20% in CEM IV 32.5 N-type cement, and the compressive strengths of the specimens were determined at 7, 28, and 90 days. Subsequently, the hydration mechanisms of specimens at different curing ages were characterized using XRD, FT-IR, DTA/TG, and SEM-EDS; in addition, their chemical and physical properties were evaluated through standard cement tests. It should be noted that olivine powder, in terms of its chemical composition, shows similarities to high-magnesium volcanic pozzolans [16,19,84]. According to the BS EN 197-1 standard, CEM IV 32.5 N type cement may contain 11–55% silica fume, pozzolan, or fly ash [85]. In preliminary trials, olivine powder was tested as a partial replacement for CEM IV 32.5 N cement at substitution levels of 30%, 40%, and 50%. However, the compressive strengths obtained at these levels did not meet the threshold values specified in the BS EN 197-1 standard [85]; therefore, the time-dependent hydration process of these ratios was not monitored. Consequently, in this study, olivine powder was used as a replacement for CEM IV 32.5 N cement at a maximum substitution level of 20%.

## 2. Materials and Methods

### 2.1. Materials

Within the scope of this study, CEM IV 32.5 N-type Portland cement obtained from the Göltaş cement factory, olivine powder sourced from the Muğla region of Turkiye, CEN standard sand, and tap water were used. The chemical properties of the CEM IV 32.5 N cement and olivine used in this study are presented in Table 2.

### 2.2. Methods

Pure and homogeneous olivine powder was sieved using an LSN-200 Hosokawa Alpine Air Jet Sieve device to obtain a 90-micron fraction for use as a cement replacement material. The specific surface areas of the olivine powder-substituted cement specimens were determined using a Toni Technik Model 7202 device, while their specific gravities were determined using a Quanta Chrome MVP-1 model device.

Mortar specimens were prepared according to BS EN 196-1 [84] standard using a laboratory-type mixer, then placed in rectangular prismatic molds measuring 40 × 40 × 160 mm and subjected to compaction. The notation and mixture information for the olivine-substituted specimens used in the study are presented in Table 3.

The prepared specimens were stored in laboratory conditions at 90% humidity and 20 ± 1 °C for 24 h. Following this period, the specimens were demoulded and maintained in a curing pool until the testing date. At 7, 28, and 90 days, specimens were retrieved from the pool and split in half, yielding six specimens. Their compressive strength was determined using a Toni Technic testing machine with a 1500 kN load capacity in accordance with the BS EN 196-1 [86] standard. The final compressive strengths were calculated by averaging the compressive strength values of the specimens.

After being subjected to compressive strength testing, the specimens were pulverized for subsequent XRD, FT-IR, DTA/TG, and SEM-EDS analyses. X-ray diffraction was conducted using a Bruker D8 Advance Twin-Twin instrument. FT-IR analysis was performed using a Jasco FT/IR 4700 Fourier Transform Infrared Spectroscopy. Thermal analyses were carried out using a Seiko SII TG/DTA 7200 instrument. SEM-EDS analyses were performed using a Fei Quanta FEG 250 instrument. The experimental procedures are summarized in Table 4.

## 3. Experimental Results and Discussion

### 3.1. Physical and Chemical Analysis

Sieve analysis, specific gravity, and Blaine-specific surface area tests were performed to determine the physical properties of the olivine-substituted specimens, and XRF tests were performed to determine the chemical properties. The physical properties of the olivine-substituted samples are presented in Table 5.

As observed in Table 4, the specific gravity increases, while the Blaine specific surface area decreases with increasing substitution rate. This phenomenon can be attributed to the higher specific gravity of olivine compared to cement. According to Table 5, the chemical composition of olivine primarily consists of CaO, Fe_2_O_3_, MgO, and SiO_2_. Various olivine powders with different chemical compositions have been reported in the literature [88,89]. This variation in chemical composition can be attributed to regional differences and geological stratification of olivine deposits. Furthermore, the MgO content in olivine is approximately 30 times higher than that in cement. It is well established that excessive MgO content in clinker leads to expansion [85]. However, in our study, olivine substitution up to 10% did not cause expansion. Previous studies in the literature have investigated the reactivity of MgO [90,91], which supports our findings.

### 3.2. XRD Analysis of the Cement Mortar

**Figure 1:**
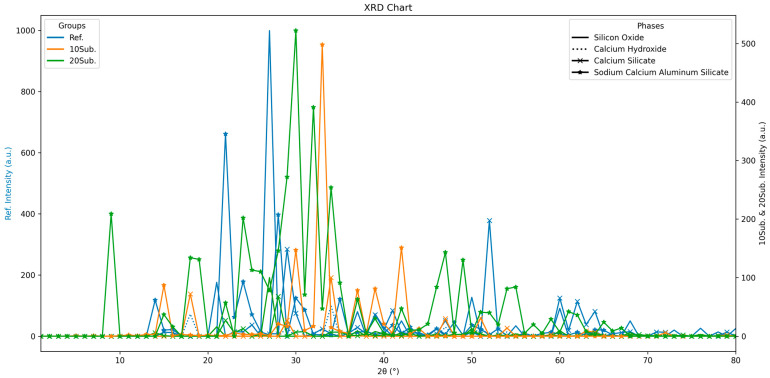
The 7-day XRD analyses of Ref, 10Sub, and 20Sub specimens.

Images showing the 7-day XRD analysis results of Ref, 10Sub, and 20Sub specimens are given in Figure 1.

**Figure 2:**
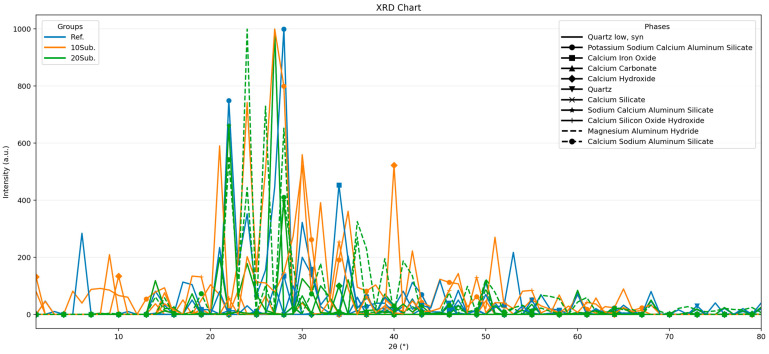
The 28-day XRD analyses of Ref, 10Sub, and 20Sub specimens.

The analysis of 7-day XRD results reveals that the reference specimen is dominated by quartz, calcium silicates, and feldspar group minerals [92]. The hydration of belite (C_2_S) and alite (C_3_S) components resulted in the formation of portlandite (Ca(OH)_2_) and calcium silicate hydrate (C-S-H) gels [93]. The distinct Ca(OH)_2_ peaks around 2 Theta 18° and 34° provide evidence for this. The Ca(OH)_2_ peaks in the 10Sub specimen are more pronounced compared to both the reference and 20Sub specimens. This indicates that 10% olivine substitution contributes to Ca(OH)_2_ formation during the hydration process. Due to the prominent larnite (Ca_2_SiO_4_) and hatrurite (Ca_3_SiO_5_) peaks, there is no adverse effect on strength performance [94]. Olivine also acted as an additional silicate source due to its magnesium silicate content [95]. However, in the 20Sub specimen, despite the release of additional silicate, Ca_2_SiO_4_ and Ca_3_SiO_5_ formations were not observed. This negatively impacted the strength. The 28-day XRD analysis results for the Ref, 10Sub, and 20Sub specimens are presented in Figure 2.

**Figure 3:**
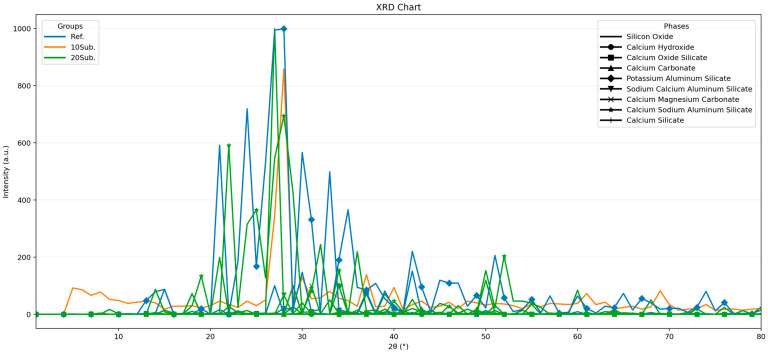
The 90-day XRD analyses of Ref, 10Sub, and 20Sub specimens.

Examination of the 28-day XRD results for the reference specimen reveals ongoing hydration ((Ca(OH)_2_, albite (NaAlSi_3_O_8_), orthoclase (KAlSi_3_O_8_), and calcium silicate (CaSiO_3_)), while unhydrated clinker phases (SiO_2_) are still present. In the XRD pattern of the 10Sub specimen, the higher Ca(OH)_2_ peak and lower SiO_2_ peak compared to the reference specimen can be attributed to the influence of olivine substitution on the hydration process. Additionally, the olivine substitution led to the formation of the calcium silicate hydroxide (Ca_5_(Si_6_O_16_)(OH)_2_) structure, resulting in a modification of hydration products. The analysis of the XRD pattern for the 20Sub specimen shows a decrease in the Ca(OH)_2_ peak compared to the 10Sub specimen. Furthermore, due to the excessive Mg content [96], magnesium aluminum hydride (Mg(AlH_4_)_2_) and anorthite (Ca_0.66_Na_0.34_Al_1.66_Si_2.34_O_8_) phases were formed. This phenomenon [97] disrupted the hydration mechanism of the 20Sub specimen, leading to a reduction in strength. The 90-day XRD analysis results for the Ref, 10Sub, and 20Sub specimens are presented in Figure 3.

Detailed examination of 90-day XRD patterns indicates that the reference specimen has largely completed its hydration (Ca(OH)_2_), although carbonation (calcite (CaCO_3_)) continues. In the 10Sub specimen, while the Ca(OH)_2_ content decreased compared to the reference specimen, slight increases in CaCO_3_ and SiO_2_ components were observed. This supported C-S-H formation without causing excessive depletion of Ca(OH)_2_, as both C-S-H and Ca(OH)_2_ formed as products of the same hydration reaction [98]. In the 20Sub specimen, the increased formation of carbonation (CaCO_3_ + H_2_O), SiO_2_, and NaK-SiAlO_8_ compared to the reference and 10Sub specimens disrupted the phase structure [99]. This resulted in increased strength loss over the long term [100].

However, olivine replacement is particularly evident with the decrease in CH peaks on days 28 and 90. This can be explained by the reaction of silicates that contribute to olivine formation. Furthermore, the increased amount of gel phases is evidence of olivine’s participation in the hydration process. However, the high amount of olivine (20Sub) also led to greater consumption of Ca(OH)_2_. Therefore, the decrease in CH can be attributed to reactions caused by the increased olivine content and the decreased cement content.

### 3.3. FT-IR Analysis of the Cement Mortar

**Figure 4:**
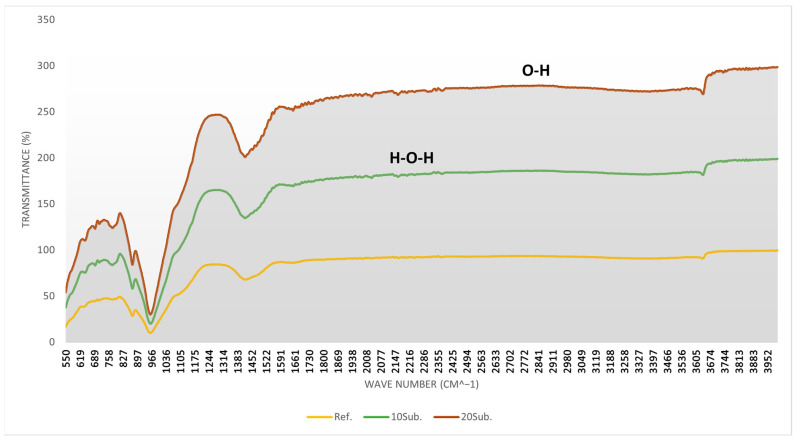
The 7-day FT-IR analyses of Ref, 10Sub, and 20Sub specimens.

**Figure 5:**
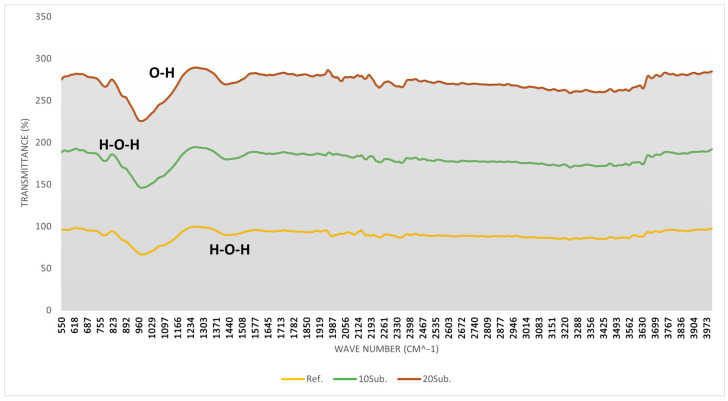
The 28-day FT-IR analyses of Ref, 10Sub, and 20Sub specimens.

**Figure 6:**
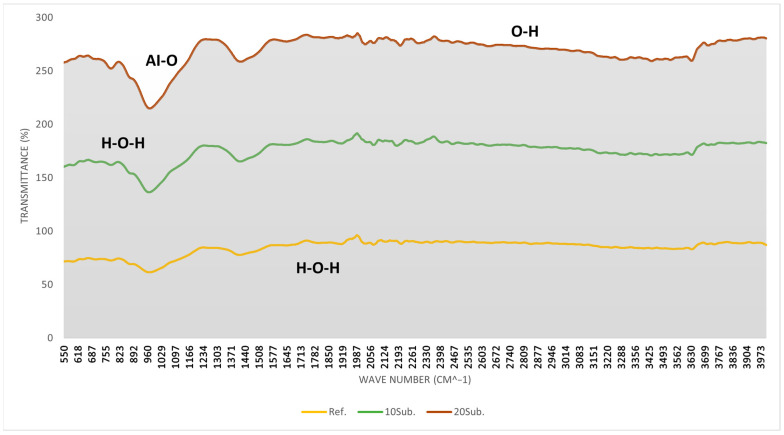
The 90-day FT-IR analyses of Ref, 10Sub, and 20Sub specimens.

In the FT-IR spectrum, the *x*-axis (Wavenumber cm^−1^) indicates how much infrared light the specimen absorbs at specific wavelengths, while the *y*-axis (Transmittance %) shows the proportion of light transmitted through the specimen [101]. In Figure 4, Figure 5 and Figure 6, the yellow line represents the reference specimen, the green line represents the 10Sub specimen, and the brown line represents the 20Sub specimen. The 7-day FT-IR analysis results for the Ref, 10Sub, and 20Sub specimens are presented in Figure 4.

The analysis of Figure 4 reveals that the more pronounced peaks in the 600–700 cm^−1^ region are attributable to increased olivine substitution rates. The increasing peak intensity in the 850–1000 cm^−1^ region correlates with higher olivine content. The weak peaks in the 1000–1700 cm^−1^ range suggest carbonation reactions between olivine and cement phases, while the prominent peaks indicate the interaction between the specimen and CO_3_^2−^-containing components (such as calcite and dolomite carbonates). The stable slope in the 2500–3200 cm^−1^ region results from cement hydration products (such as Ca(OH)_2_) or their reaction with water. Water content particularly varies in the 3200–3600 cm^−1^ region. Consequently, as the substitution rate increases, hydration products decrease, leading to increased permeability. This corresponds with the increased distance from the x-axis with higher substitution rates, as shown in Figure 4. The 28-day FT-IR analysis results for the Ref, 10Sub, and 20Sub specimens are presented in Figure 5.

As olivine is a silicate-based mineral, silicon–oxygen (Si-O), magnesium–oxygen (Mg-O), and iron–oxygen (Fe-O) bonds are particularly notable in the Figure 5 spectrum. The 500–700 cm^−1^ range indicates the reorganization of magnesium and iron bonds (Mg-O and Fe-O) in the cement matrix. Calcite formation is observed around 900 cm^−1^. Asymmetric stretching in the 10Sub and 20Sub specimens between 1000 and 1200 cm^−1^ is associated with silicon–oxygen–silicon (Si-O-Si) stretching. The peaks in this region indicate the formation of new silicate phases. Carbonation formation in the 1400–1500 cm^−1^ region can be attributed to pH changes resulting from olivine’s reaction with water. Carbonation is most pronounced in the 20Sub specimen. The formation of different peaks in the 3200–3600 cm^−1^ range for 10Sub and 20Sub specimens is related to olivine substitution affecting the water-binding mechanism in the cement matrix. An analysis of Figure 4 shows that the more pronounced peaks in the 600–700 cm^−1^ region are due to increased olivine substitution rates. The intensity of peaks in the 850–1000 cm^−1^ region correlates with increased olivine content. Weak peaks in the 1000–1700 cm^−1^ range suggest carbonation reactions between olivine and cement phases, while prominent peaks indicate interaction with CO_3_^2−^-containing components (such as calcite and dolomite carbonates). The stable slope in the 2500–3200 cm^−1^ region results from cement hydration products (such as Ca(OH)_2_) or their reaction with water. Water content particularly varies in the 3200–3600 cm^−1^ region. Consequently, as the substitution rate increases, hydration products decrease, leading to increased permeability. This corresponds with the increased distance from the x-axis with higher substitution rates, as shown in Figure 4. The 90-day FT-IR analysis results for the Ref, 10Sub, and 20Sub specimens are presented in Figure 6.

In the 90-day FT-IR spectrum (Figure 6), the 500–700 cm^−1^ range is notable as the region where olivine reacts with cement hydration products. The asymmetric stretching in the 1000–1200 cm^−1^ region is similar to the 28-day results (Figure 5). The variation between 10Sub and 20Sub specimens in this region indicates that olivine substitution affects the silicate compositions in the concrete matrix. The more pronounced peaks of the 20Sub specimen in the 3000–3600 cm^−1^ range indicate adverse effects on hydration. This observation aligns with the XRD results.

### 3.4. Thermal Analysis of the Cement Mortar

**Figure 7:**
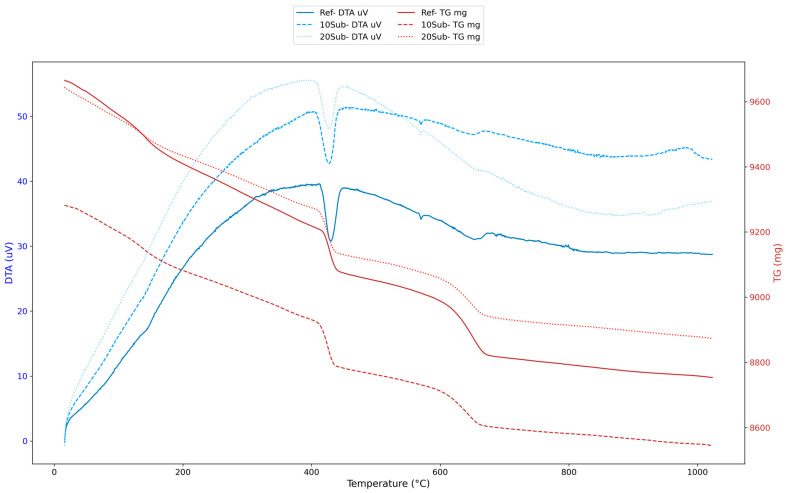
The 7-day thermal analyses of Ref, 10Sub, and 20Sub specimens.

DTA/TG analyses were conducted to understand the high-temperature resistance, hydration products, carbonation process, and phase transitions of olivine-substituted specimens. The 7-day thermal analysis results for the Ref, 10Sub, and 20Sub specimens are presented in Figure 7.

The analysis of Figure 7 reveals endothermic and exothermic reactions between 100 and 600 °C in the DTA curves. It is observed that free water evaporates between 100 and 200 °C, the C-S-H structure begins to deteriorate between 200 and 400 °C, Ca(OH)_2_ decomposition occurs between 400 and 550 °C [102] (Equation (1), and the curves become more stable after 600 °C).

Ca(OH)_2_ → CaO + H_2_O (1)

**Figure 8:**
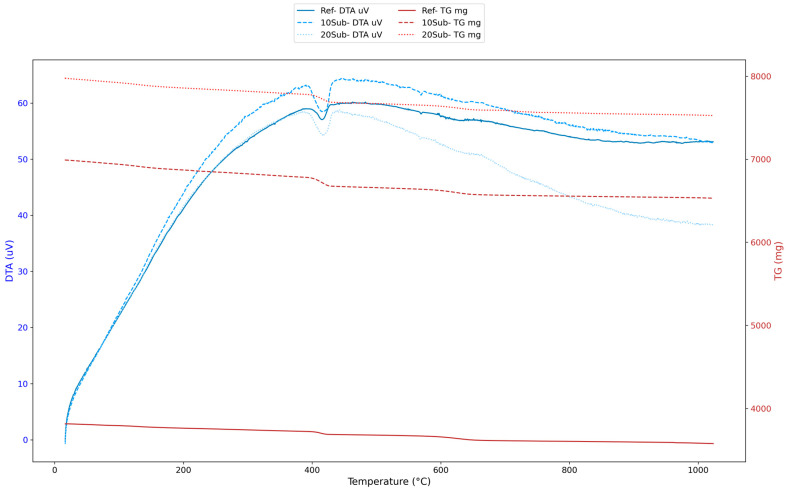
The 28-day thermal analyses of Ref, 10Sub, and 20Sub specimens.

The TG curves indicate that specimens undergo mass loss as temperature increases (particularly between 200 and 600 °C), with mass loss beginning to decelerate after 600 °C. Moreover, fewer thermal events occurred in the reference specimen. However, the 10Sub specimen is more stable due to its minimal mass loss. In the 20Sub specimen, the increased olivine substitution rate led to more pronounced phase transformations and decomposition events. The 28-day thermal analysis results for the Ref, 10Sub, and 20Sub specimens are presented in Figure 8.

Figure 8 shows that relative humidity evaporation causes negligible mass loss in the 0–200 °C range. The sharp decline in the TG curve indicates that olivine begins to decompose in the 200–600 °C range. Within this temperature range, Fe-containing olivine continued to form iron (Fe_2_O), while Mg-containing olivine formed Mg_2_O [103]. At temperatures above 800 °C, olivine completely decomposed, forming MgO, SiO_2_, and Fe_2_O_3_ [104]. Additionally, the reference specimen generally exhibited lower mass loss and thermal reaction. Although the 20Sub specimen showed the lowest thermal stability, it experienced the highest mass loss. This can be attributed to olivine’s lower thermal reactivity and the decomposition caused by excess MgO in the olivine content. This supports the formation of brucite (Mg(OH)_2_). The 10Sub specimen demonstrated moderate effects. In order to better interpret the thermal behavior of the 28-day specimens, the total mass loss between 16.49 °C and 1023.30 °C was calculated using the TG data of each specimen. The results presented in Table 6 indicate that all specimens underwent gradual decomposition, with mass losses ranging from 5.64% to 6.58%.

**Figure 9:**
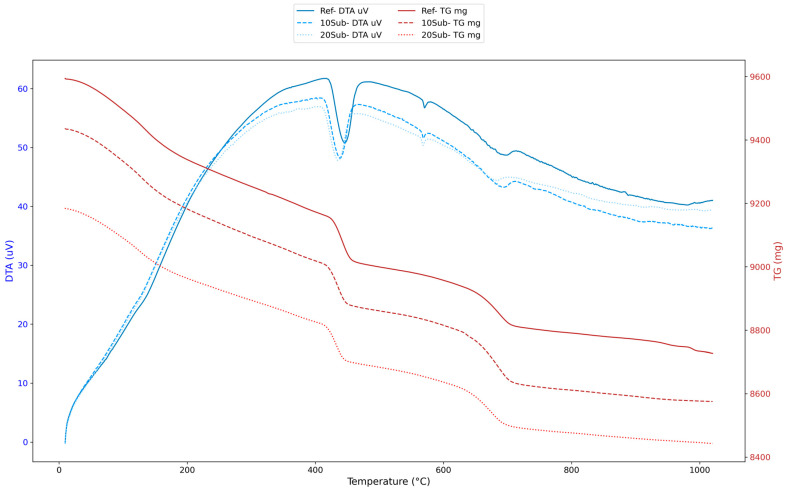
The 90-day thermal analysis of Ref, 10Sub, and 20Sub specimens.

The 20Sub sample exhibited lower total mass loss compared to both the reference and 10Sub samples. Increasing the olivine substitution rate resulted in reduced porosity and microstructural improvements. The 90-day thermal analysis results for the Ref, 10Sub, and 20Sub specimens are presented in Figure 9.

Analysis of Figure 9 reveals negligible mass losses in the 0–200 °C range. The largest endothermic peak and greatest weight loss occurred between 400 and 600 °C. Olivine began to decompose within this temperature range. Particularly, the crystal structures of Fe_2_SiO_4_ (fayalite) and Mg_2_SiO_4_ (forsterite) deteriorated [103] (Equations (2) and (3)).

Fe_2_SiO_4_ → 2FeO + SiO_2_ (2)

Mg_2_SiO_4_ → 2MgO + SiO_2_ (3)

In the 600–800 °C range, olivine completely decomposed. After 800 °C, Mg stabilized, and FeO transformed into Fe_2_O_3_ [104]. Moreover, olivine-substituted specimens appear to have lower thermal stability but greater mass loss stability compared to the reference specimen. This indicates that olivine substitution improves the long-term thermal stability of concrete by reducing mass loss.

### 3.5. SEM and EDS Analysis of the Cement Mortar

**Figure 10:**
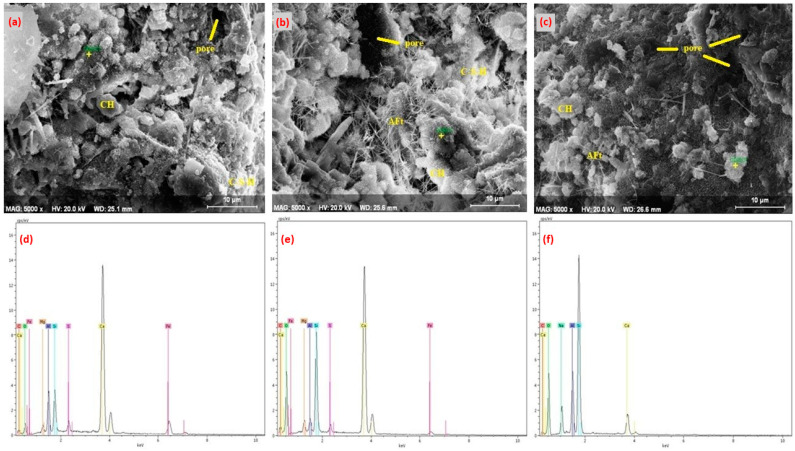
The 7-day SEM and EDS analysis of Ref (**a**,**d**), 10Sub (**b**,**e**), and 20Sub (**c**,**f**) specimens.

The 7-day SEM and EDS images of the Ref, 10Sub, and 20Sub specimens are presented in Figure 10, while the corresponding 7-day EDS results are provided in Table 7.

**Figure 11:**
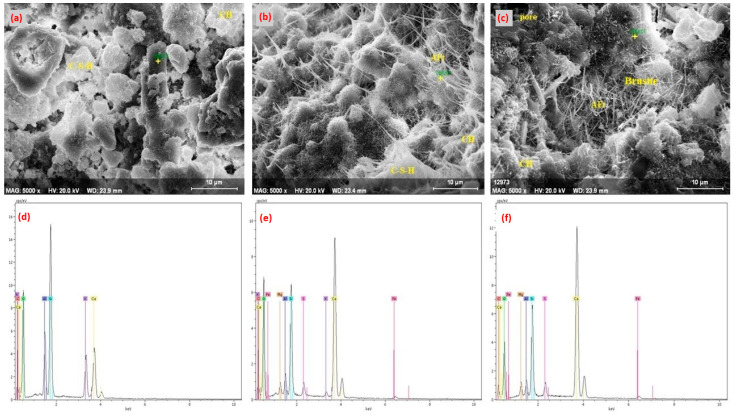
The 28-day SEM and EDS analysis of Ref (**a**,**d**), 10Sub (**b**,**e**), and 20Sub (**c**,**f**) specimens.

Figure 10a,d shows the porous and heterogeneous microstructure of the reference specimen. The C-S-H phase and Ca(OH)_2_ crystals formed after hydration are prominent. The highest peak originates from the C-S-H phase and Ca(OH)_2_. The high silicon (Si) content and low Mg and Fe ratios are attributable to the cement type. In Figure 10b,e, needle-like structures (ettringite) and a differentiated microstructure due to olivine mineral were observed. These angular particles serve as fillers within the cement matrix. Additionally, increases in Mg and Si elements are notable due to olivine content. This increase contributed to the cement matrix. The calcium (Ca) ratio is relatively lower due to the reduction in total cement content. However, the C-S-H phase still contributes to matrix durability. Figure 10c,f shows a denser microstructure and residues related to olivine minerals. Furthermore, with olivine substitution, the increase in Mg and Si ratios and decrease in Ca ratio continued. The increase in olivine content slowed hydration. Studies in the literature demonstrate similar situations causing hydration retardation [105,106,107,108,109,110,111]. The 28-day SEM and EDS images of the Ref, 10Sub, and 20Sub specimens are presented in Figure 11, while the corresponding 28-day EDS results are provided in Table 8.

**Figure 12:**
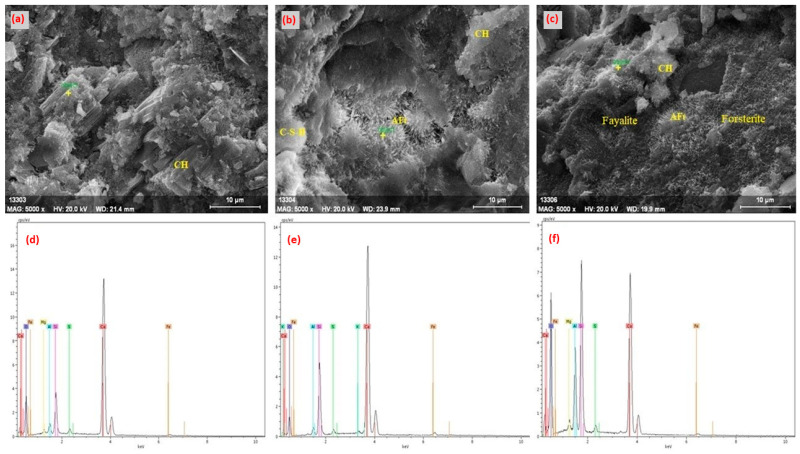
The 90-day SEM and EDS analysis of Ref (**a**,**d**), 10Sub (**b**,**e**), and 20Sub (**c**,**f**) specimens.

The examination of 28-day SEM images of the reference specimen (Figure 11a,d) shows an increase in classical C-S-H phases that enhance strength properties. Additionally, the presence of large and irregular crystal structures (Ca(OH)_2_) is notable. This limits long-term strength development due to carbonation risk. The 10Sub specimen (Figure 11b,e) exhibits a more compact microstructure compared to the reference specimen (Figure 11a,d). Olivine particles have integrated more tightly and densely into the cement matrix. This is related to both the physical filling effect and the chemical properties of olivine. The Mg content from olivine facilitated increased silicate formation in hydration products, manifesting in needle-like structures. While the 20Sub specimen (Figure 11c,f) exhibits a dense microstructure, it shows issues with homogeneity. More pronounced clustering of olivine particles within the matrix has led to irregularities and heterogeneous structures. This results from the high presence of Mg (Brucite formation). The density of C-S-H phases increased but displayed a less homogeneous structure compared to the 10Sub specimen (Figure 11b,e). The 90-day SEM and EDS images of the Ref, 10Sub, and 20Sub specimens are presented in Figure 12, while the corresponding 90-day EDS results are provided in Table 9.

At the end of the 90-day hydration, mature C-S-H phases and Ca(OH)_2_ crystals are clearly observed in the reference specimen (Figure 12a,d). Although most pores have closed, capillary voids still persist in some regions. This formation aligns with the expected element distribution for the reference specimen. In the cement matrix formed with 10% olivine substitution, a more compact structure was observed after the 90-day period (Figure 12b,e). The homogeneous distribution in the chemical structure enhanced the microstructure’s strength [112]. Although Ca content decreased due to olivine substitution, it did not lead to significant weakening in the microstructure. Furthermore, olivine contribution strengthened the microstructure by promoting the formation of Mg-containing phases. The 90-day curing period largely eliminated pores, contributing to matrix homogenization. This indicates optimization of the hydration process through olivine contribution. A 20% olivine substitution increased the density but limited void filling in some regions (Figure 12c,f). Fayalite and forsterite decomposition occurred. The density of hydration products indicates that olivine contribution did not have a strong effect on binding phases. Although the decrease in the Ca content supported the formation of new structures incorporating olivine contribution in binding phases, regional heterogeneity led to strength reductions. Additionally, a significant decrease in the Ca ratio was observed with increased olivine content. This resulted in slower hydration processes.

### 3.6. Compressive Strength

**Figure 13:**
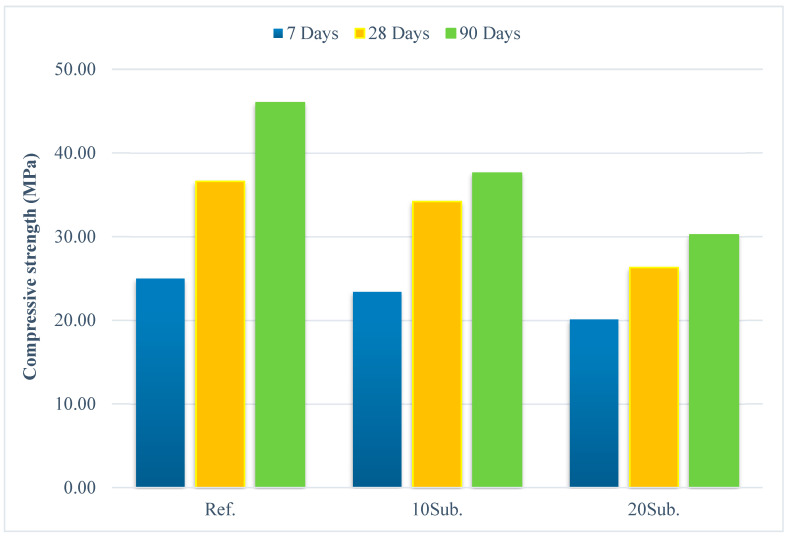
The 7, 28, and 90-day compressive strength values of Ref, 10Sub, and 20Sub specimens.

The 7-, 28-, and 90-day compressive strength values for Ref, 10Sub, and 20Sub specimens are presented in Figure 13.

The compressive strength of the cement mortar depends on the mixing-water content, hydration process, specific surface area of the binding material, substitution rate, and granulometry [36,113,114]. An analysis of Figure 13 shows that the compressive strength reduction rates of 10Sub specimens compared to the reference specimen are as follows: 6.40% for 7-day specimens, 6.56% for 28-day specimens, and 18.26% for 90-day specimens. The compressive strength reduction rates of 20Sub specimens compared to the reference specimen are as follows: 19.60% for 7-day specimens, 28.14% for 28-day specimens, and 34.35% for 90-day specimens. The data obtained indicates that the compressive strength decreases with the increasing substitution rate. SEM images (Figure 10, Figure 11 and Figure 12) support that the 20Sub specimen contains more voids compared to the reference and 10Sub specimens. Although the compressive strength value of the 10Sub specimen is lower than the reference specimen, it remains within the limit values according to BS EN 197-1 [85] standard. This indicates that olivine can be substituted for CEM IV 325 N-type cement up to 10%.

## 4. Conclusions

In this study investigating the hydration mechanisms of olivine-substituted cement mortars, the role of olivine on compressive strength, microstructural characteristics, and physical properties was examined in detail. The study concluded that using olivine as a primary raw material in cement would be beneficial from both environmental and economic perspectives. In particular, reducing the carbon footprint will contribute to making cement a more environmentally friendly and sustainable building material. In this context, the results obtained from the study are as follows:

- Cement is the phase responsible for strength development.
- Olivine fine fractions fill voids through a filling effect, increasing the density of the matrix.
- According to XRD results, while the 20Sub specimen exhibited a more porous structure, the 10Sub specimen contained higher concentrations of Ca(OH)_2_ and C-S-H phases.
- The olivine substitution significantly altered the chemical bonds of Si-O, Mg-O, and Fe-O in the cement matrix, leading to notable changes in the FT-IR spectrum. This indicates that 10% olivine substitution positively influenced the hydration and carbonation processes.
- DTA/TG analysis revealed that 20% olivine substitution decreased the thermal stability of concrete while effectively reducing mass loss. The 10% olivine substitution demonstrated a moderating effect, producing intermediate results for both parameters. The reference specimen exhibited the highest values for both parameters. These findings suggest that olivine substitution has the potential to enhance the long-term properties of concrete, although higher substitution rates may compromise certain characteristics.
- SEM-EDS analysis indicated that olivine substitution improved the microstructure of cement. However, incorporating olivine beyond 10% adversely affected the homogeneity and strength of the matrix.
- The 10% olivine substitution strengthened the cement matrix by facilitating the balanced formation of microstructure and hydration products.
- In contrast, 20% olivine substitution presented a heterogeneous structure in the cement matrix and negatively impacted hydration.
- The compressive strength value of the 10Sub specimen remains within the limit values specified by BS EN 197-1 [85] standard.
- Olivine can be substituted for CEM IV 32.5 N-type cement up to 10%.
- The ability to use olivine directly as a cement replacement material without calcination shows promise for green cement and ecological concrete production. The results demonstrate the viability of olivine substitution in sustainable building materials and its effects on microstructure.

In conclusion, olivine substitution alters the chemical structure of concrete, particularly through interactions with silicate, carbonate, and oxide groups. This study demonstrates that concrete containing olivine can have significant effects on environmental durability and mechanical properties.

## 5. Recommendations

While olivine substitution may result in lower early-age strength, this can be addressed through careful monitoring of the curing process and implementation of extended curing conditions.

As the olivine content increases, the Ca ratio in the cement matrix decreases. Therefore, combinations with different additives should be developed, particularly to balance the Ca content. This approach could enhance the homogeneity and strength of the matrix.

The incorporation of olivine improves the thermal reactions and structural properties of olivine-substituted specimens. This suggests that olivine substitution could be particularly advantageous in applications where the long-term thermal stability of concrete is critical. Additionally, olivine can have beneficial effects on water permeability by filling voids in the concrete microstructure. In this context, the use of olivine could contribute to efforts in developing sustainable construction materials. Therefore, further research into the utilization of olivine in cement and concrete production would be beneficial.

## Figures and Tables

**Table 1:** Mix proportions of cement mortars with olivine substitution.

Purpose	Results	Researcher(s)
They investigated the usability of olivine aggregate in calcium lime mortars.	They determined that olivine aggregate reacts with lime and carbon dioxide in humid environments to form dolomite within the mortar, thereby improving mechanical properties and increasing CO_2_ absorption capacity.	[15]
They studied the CO_2_ capture efficiency of olivine aggregate in cement and lime mortars from the atmosphere.	They found that between lime and cement mortars, lime mortars captured more CO_2_ and produced stable compounds.	[18]
They researched the feasibility of utilizing volcanic slag in cement.	They indicated that olivine and other mineralogical components in volcanic slag contained high levels of magnesium oxide (MgO), hematite (Fe_2_O_3_), calcium oxide (CaO), and titanium dioxide (TiO_2_), suggesting that volcanic slag less exposed to weathering conditions would be suitable as an additive in cement production.	[19]
They characterized lightweight, olivine nano-silica-activated slag-fly ash composites.	They discovered that using olivine nano-silica as an activator reduced carbon emissions by approximately 25%.	[20]
They examined the performance of olivine in environments with low and high pH values.	They determined that olivine enhances the leakage resistance of well wall cement through carbonation (CSH and CH), reacts with hydrochloric acid (HCl) aqueous solution at pH values between 1.0 and 1.92, and shows no significant reactivity in other liquids.	[21]
They investigated the effects of olivine nano-silica addition on the mixture stability, rheology, and hydration degree of oil well cement.	They found that olivine nano-silica accelerates oil well cement reactions and enhances mechanical properties.	[22]
They studied the CO_2_ sequestration efficiency of Mg^2+^ ions obtained through olivine dissolution.	They established that olivine applications would provide limited benefits during carbon remediation strategies, while the use of magnesium (Mg)-based cement would increase efficiency.	[23]
They researched the potential use of silica recovered from olivine through an acid digestion process as an additive in cement.	They determined that silica recovered from olivine improves the binding properties of cement and provides significant environmental benefits.	[24]
They examined the behavior of mineral-added mortars under high-temperature conditions.	They found that across all temperature values, the best results were obtained from specimens containing 10% basalt, pyrophyllite, and olivine.	[25]
They developed and characterized the material properties of a highly dispersed colloidal olivine nano-silica (C-OnS).	They discovered that C-OnS enhances the early-age performance of ultra-high-performance concrete due to its high silanol content, surface area, and dispersity.	[26]

**Table 2:** Chemical analysis results of produced materials.

Specimen	CaO (%)	Fe_2_O_3_ (%)	Al_2_O_3_ (%)	MgO (%)	Na_2_O (%)	K_2_O (%)	SiO_2_ (%)	SO_3_ (%)
Olivine	3.18	9.45	-	46.20	-	-	38.14	-
CEM IV 32.5 N	62.57	2.56	4.60	1.53	0.26	0.66	20.36	3.32

**Table 3:** Notation and blend information for olivine-substituted specimens.

Notation	Substitution Rates (%)	Water (g)	Cement (g)	Olivine (g)	Standard Sand (g)
Ref	0	225	450	0	1350
10Sub	10		405	45	
20Sub	20		360	90	

**Table 4:** Experimental study data.

Experiment	Parameter	Standard
Sieve analysis	Classification according to material size	[87]
Specific surface area (Blaine)	Specific surface area	[87]
Specific weight	Specific weight	
Compressive strength	Compression	[86]
XRD	Mineral phase analysis	
FT-IR	Material identification and verification	
DTA-TG	Thermal analysis	
SEM	Surface morphology	
EDS	Chemical composition	

**Table 5:** Physical properties of the specimens.

Notation	Grain Size >45 μm >90 μm (%)		Specific Gravity (g/cm^3^)	Blaine Specific Surface Area (cm^2^/g)
Ref	0.0	1.2	2.95	3822
10Sub	2.5	1.1	3.11	3720
20Sub	3.8	1.7	3.11	3550

**Table 6:** Mass Loss of specimens between 16.5 °C and 1023.3 °C.

Notation	TG Start (mg)	TG End (mg)	Mass Loss (mg)	Mass Loss (%)
Ref	3815.15	3577.95	237.20	6.22
10Sub	6991.51	6531.44	460.07	6.58
20Sub	7976.78	7526.51	450.27	5.64

**Table 7:** The 7-day EDS data of Ref, 10Sub, and 20Sub specimens.

	Reference				10Sub				20Sub			
Element	Weight	Atomic	Oxide		Weight	Atomic	Oxide		Weight	Atomic	Oxide	
	(%)	(%)	(%)		(%)	(%)	(%)		(%)	(%)	(%)	
C	4.03	4.78	CO_2_	14.62	3.51	3.19	CO_2_	12.87	3.00	2.77	CO_2_	9.56
Mg	0.76	0.90	-	0.75	2.52	2.29	-	2.53	-	-	-	-
Na	-	-	-	-	-	-	-	-	6.90	6.36	Na_2_O	8.08
Al	5.66	6.73	Al_2_O_3_	10.60	2.21	2.00	Al_2_O_3_	4.17	10.46	9.65	Al_2_O_3_	17.17
Si	5.81	6.91	SiO_2_	12.32	13.79	12.53	SiO_2_	29.55	31.69	29.22	SiO_2_	58.87
S	1.32	1.57	SO_3_	3.27	1.29	1.17	SO_3_	3.21	-	-	-	-
Ca	33.85	40.22	CaO	46.94	31.76	28.86	CaO	44.49	5.20	4.80	CaO	6.32
Fe	8.11	9.64	Fe_2_O_3_	11.50	2.22	2.02	Fe_2_O_3_	3.18	-	-	-	-
O	24.63	29.26	-	31.18	52.75	47.94	-	12.37	51.20	47.21	-	7.75

**Table 8:** The 28-day EDS data of Ref, 10Sub, and 20Sub specimens.

	Reference				10Sub				20Sub			
Element	Weight	Atomic	Oxide		Weight	Atomic	Oxide		Weight	Atomic	Oxide	
	(%)	(%)	(%)		(%)	(%)	(%)		(%)	(%)	(%)	
C	3.07	2.87	CO_2_	14.16	1.34	1.17	CO_2_	6.08	5.59	5.48	CO_2_	22.26
Mg	-	-	-	-	2.27	1.98	-	2.82	1.95	1.92	-	2.12
Al	4.80	4.49	Al_2_O_3_	11.41	2.67	2.33	Al_2_O_3_	6.25	1.74	1.71	Al_2_O_3_	3.58
Si	15.76	14.72	SiO_2_	42.38	12.16	10.61	SiO_2_	32.26	9.86	9.67	SiO_2_	22.93
S	-	-	-	-	1.62	1.41	SO_3_	5.01	1.54	1.51	SO_3_	4.19
K	7.75	7.23	K_2_O	11.73	0.52	0.27	K_2_O	0.90	-	-	-	-
Ca	11.55	10.78	CaO	20.31	25.67	22.40	CaO	44.54	28.43	27.88	CaO	43.23
Fe	-	-	-	-	1.05	0.38	Fe_2_O_3_	2.14	1.08	1.06	Fe_2_O_3_	1.68
O	64.15	59.91	-	31.35	67.05	58.51	-	37.51	51.77	50.77	-	12.52

**Table 9:** The 90-day EDS data of Ref, 10Sub, and 20Sub specimens.

	Reference				10Sub				20Sub			
Element	Weight	Atomic	Oxide		Weight	Atomic	Oxide		Weight	Atomic	Oxide	
	(%)	(%)	(%)		(%)	(%)	(%)		(%)	(%)	(%)	
Mg	1.03	0.99	-	1.35	-	-	-	-	1.81	1.57	-	2.03
Al	1.79	1.71	Al_2_O_3_	4.41	0.15	0.17	Al_2_O_3_	0.36	8.19	7.07	Al_2_O_3_	17.33
Si	6.83	6.51	SiO_2_	19.04	9.34	10.21	SiO_2_	24.65	16.68	14.40	SiO_2_	39.98
S	0.81	0.78	SO_3_	2.65	0.16	0.18	SO_3_	0.50	0.82	0.71	SO_3_	2.30
K	-	-	-	-	0.62	0.68	K_2_O	0.92	-	-	-	-
Ca	38.78	36.99	CaO	70.70	41.26	45.11	CaO	71.21	23.69	20.46	CaO	37.14
Fe	1.00	0.96	Fe_2_O_3_	1.87	1.34	1.47	Fe_2_O_3_	2.36	0.76	0.66	Fe_2_O_3_	1.22
O	54.59	52.07	-	36.76	38.59	42.19	-	16.98	63.85	55.14	-	29.67

## Data Availability

The original contributions presented in this study are included in the article. Further inquiries can be directed to the corresponding author.
